# Diabetes and Breast Cancer Subtypes

**DOI:** 10.1371/journal.pone.0170084

**Published:** 2017-01-11

**Authors:** Heleen K. Bronsveld, Vibeke Jensen, Pernille Vahl, Marie L. De Bruin, Sten Cornelissen, Joyce Sanders, Anssi Auvinen, Jari Haukka, Morten Andersen, Peter Vestergaard, Marjanka K. Schmidt

**Affiliations:** 1 Division of Molecular Pathology, The Netherlands Cancer Institute, Amsterdam, Netherlands; 2 Division of Psychosocial Research and Epidemiology, The Netherlands Cancer Institute, Amsterdam, Netherlands; 3 Department of Pathology, Aarhus University Hospital THG, Aarhus, Denmark; 4 Division of Pharmacoepidemiology & Clinical Pharmacology, Utrecht University, Utrecht, Netherlands; 5 Division of Pathology, The Netherlands Cancer Institute, Amsterdam, Netherlands; 6 School of Public Health, University of Tampere, Tampere, Finland; 7 Department of Public Health, University of Helsinki, Helsinki, Finland; 8 Department of Medicine, Karolinska Institute, Stockholm, Sweden; 9 Clinical Institute, Aalborg University, Aalborg, Denmark; University of South Alabama Mitchell Cancer Institute, UNITED STATES

## Abstract

**Background:**

Women with diabetes have a worse survival after breast cancer diagnosis compared to women without diabetes. This may be due to a different etiological profile, leading to the development of more aggressive breast cancer subtypes. Our aim was to investigate whether insulin and non-insulin treated women with diabetes develop specific clinicopathological breast cancer subtypes compared to women without diabetes.

**Methods and Findings:**

This cross-sectional study included randomly selected patients with invasive breast cancer diagnosed in 2000–2010. Stratified by age at breast cancer diagnosis (≤50 and >50 years), women with diabetes were 2:1 frequency-matched on year of birth and age at breast cancer diagnosis (both in 10-year categories) to women without diabetes, to select ~300 patients with tumor tissue available. Tumor MicroArrays were stained by immunohistochemistry for estrogen and progesterone receptor (ER, PR), HER2, Ki67, CK5/6, CK14, and p63. A pathologist scored all stains and revised morphology and grade. Associations between diabetes/insulin treatment and clinicopathological subtypes were analyzed using multivariable logistic regression. Morphology and grade were not significantly different between women with diabetes (n = 211) and women without diabetes (n = 101), irrespective of menopausal status. Premenopausal women with diabetes tended to have more often PR-negative (OR = 2.44(95%CI:1.07–5.55)), HER2-negative (OR = 2.84(95%CI:1.11–7.22)), and basal-like (OR = 3.14(95%CI:1.03–9.60) tumors than the women without diabetes, with non-significantly increased frequencies of ER-negative (OR = 2.48(95%CI:0.95–6.45)) and triple negative (OR = 2.60(95%CI:0.88–7.67) tumors. After adjustment for age and BMI, the associations remained similar in size but less significant. We observed no evidence for associations of clinicopathological subtypes with diabetes in postmenopausal women, or with insulin treatment in general.

**Conclusions:**

We found no compelling evidence that women with diabetes, treated with or without insulin, develop different breast cancer subtypes than women without diabetes. However, premenopausal women with diabetes tended to develop breast tumors that do not express hormonal receptors, which are typically associated with poor prognosis.

## Introduction

Diabetes mellitus and breast cancer are chronic diseases with increasing incidence in many countries [1,2]. Recent estimates indicate that diabetes prevalence is 9.1% among women in Europe [1] and life-time risk for breast cancer is 9.7% [3]. Most patients with diabetes (~90%) have type 2 disease, characterized by reduced insulin secretion and insulin resistance with diagnosis in late adulthood, while patients with type 1 diabetes are insulin deficient [4].

Several studies have investigated whether diabetes and/or insulin (analogue) treatment increase breast cancer risk [5–10] or affect prognosis [11–18], because of their potential impact on tumor progression through e.g. the insulin-like growth receptor pathway [5,19]. Women with diabetes have a 15–20% increased risk of breast cancer compared to women without diabetes [6–9], but no impact of insulin analogue treatment has been shown [5]. Breast cancer in women with diabetes is often diagnosed at an advanced stage compared to women without diabetes [13,14,20–22]. Moreover, overall mortality after breast cancer diagnosis has been shown to be 30–60% higher in women with diabetes compared to women without diabetes [11–16], even after adjustment for tumor stage [13,14,16]. However, studies that investigated the association between breast cancer-specific mortality and diabetes show inconsistent results [11,23–27].

Diabetes itself might have a direct effect on breast cancer prognosis due to physiological effects of hyperglycemia, or hyperinsulinemia, which is a hallmark of insulin resistance commonly observed in patients with type 2 diabetes [28,29]. It has been shown that cancer-specific survival was decreased for women with abnormal glycemic status [25,27] and that fasting insulin levels are associated with worse outcome (distant recurrence and death), independent of Body Mass Index (BMI) [30]. However, diabetes itself and its complications may also increase risk of overall mortality [4] and shared cancer-promoting factors in patients with diabetes, such as obesity and a sedentary lifestyle, increases also the risk of death from competing causes (metabolic/cardiovascular diseases).

Another reason for the worse breast cancer survival may be that women with diabetes develop a more aggressive or less treatment-responsive tumor subtype. It has already been shown that hormone-related breast cancer and diabetes risk factors, such as obesity, are associated with the development of ER-negative breast cancer subtypes [31,32]. Insulin interacts with estrogens; there is experimental support that insulin may enhance estrogen production, stimulating the development of ER-positive breast cancer [19]. Furthermore, the promotion of tumor cell growth upon insulin exposure may differ by breast cancer subtype; we know from *in vitro* studies that mitogenic potential of insulins depends on the type of breast cancer cell line [5,33]. Although breast cancer subtypes have been extensively studied in the general population [31], few studies have assessed breast cancer subtypes in women with diabetes.

The aim of this study is to determine whether breast cancer patients with diabetes have a specific clinicopathological tumor subtype compared to those without diabetes, and whether the use of insulin is related to this.

## Methods

The study protocol was approved by the Science Ethics Committee of the Region Midtjylland in Denmark. The Science Ethics Committee of the Region Midtjylland in Denmark approved that informed consent for this study was not obtained; however, all women had the possibility to opt-out from research through the nation-wide registry. Tumor tissue of the women had been collected for diagnostic or therapeutic purposes around the time of breast cancer diagnosis. This tissue is stored in biobanks and may be used for research (‘secondary use’) as long as coded and anonymous to the researcher. No tissue was used against the will of the patients (women who opt-out with regard to tissue use for future scientific purposes were excluded (http://sundhedsdatastyrelsen.dk/da/registre-og-services/vaevsanvendelsesregisteret); no risk was posed to the women as the tissue had already been removed; and tumor tissue and data were anonymous for the researcher.

### Study design and patient selection

**Fig 1:**
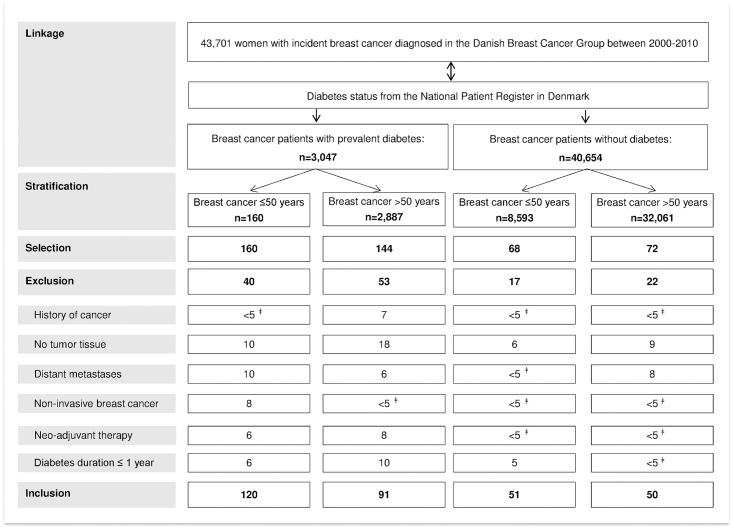
Flow chart of patient identification and selection. Stratified by age at breast cancer diagnosis (≤50 and >50 years), women with diabetes were 2:1 frequency-matched on year of birth and age at breast cancer diagnosis (both in 10-year categories) to women without diabetes, to select ~300 patients with tumor tissue available. ^ǂ^ Exact numbers <5 cannot be shown according to regulations of Statistics Denmark.

The study population consists of Caucasian women with and without diabetes, diagnosed with primary breast cancer between 2000 and 2010. The breast cancer patients were selected from a previously established nation-wide hospital-based cohort, by the Danish Breast Cancer Cooperative Group (DBCG) [34]. This cohort was linked to the National Patient Register in Denmark to identify women with and without diabetes, covering the years since 1977. In total, 43,701 women were diagnosed with incident breast cancer in 2000–2010 in the DBCG, of whom 3,047 had diabetes (7.0%). We used a cross-sectional study design with a randomly selected target population of 300 breast cancer patients. The selected women included breast cancer patients with diabetes (exposed) and without diabetes (non-exposed) sampled as follows: a random sample of women with diabetes in strata of age ≤50 and >50 years (1:1) at breast cancer diagnosis (stratification by age to increase the number of young women) frequency matched with women without diabetes from the same database (1:2) by year of birth and age at diagnosis (both in 10-year categories) (Fig 1). Twice as many women with diabetes were included as women without diabetes to allow analyses by insulin treatment. Patients with a history of other cancers, non-invasive or metastasized breast cancer, those treated with neo-adjuvant therapy, patients with diabetes diagnosed ≤1 year prior to their breast cancer diagnosis, and patients with no or insufficient tumor tissue were excluded.

### Data collection

Age, menopausal status, year of breast cancer diagnosis and information on tumor and tumor treatment were obtained from the DBCG databank and the pathology register of the women. Only age, year of breast cancer diagnosis, and diabetes status were available at the time of patient identification. Diabetes status, diabetes type (1 or 2), and age at diabetes diagnosis, as well as data on socioeconomic status were collected by linkage with the National Patient Register (which included all medical diagnoses from 1977 onwards) in Denmark. Data on medication use, available from 1995 onwards, was obtained by linkage with the Danish Register of Medicinal Products Statistics. All linkages were done using codes which render the data anonymous to the researchers who do not have direct access to these source databases. Women were defined as oral contraceptive or hormone replacement users if at least 2 prescriptions of the drug were prescribed cumulatively in the period up to one year prior to breast cancer diagnosis. Additional information on height, weight, Body Mass Index (BMI), smoking, alcohol use, and HbA_1_C levels (measure of average glucose levels) prior to breast cancer diagnosis were retrieved from electronic patient files and anonymized before inclusion in the database for the researchers. Formalin-fixed, paraffin-embedded tissue samples of the primary tumors were retrieved from different Departments of Pathology in Denmark, for central pathology review and immunohistochemical (IHC) analyses.

### Tumor review and IHC analyses

All formalin-fixed, paraffin-embedded tumors blocks of the primary tumor of each patient were collected and whole slides were stained with Hematoxylin and Eosin. The most representative tumor block was selected for the analyses. Hematoxylin and Eosin slides were reviewed by a breast pathologist for morphology and grade (VJ). Grade was scored following the modified Bloom-Richardson system.

For the IHC analysis, tissue microarrays with 2 cores of 2 mm per tissue block were constructed. Tissue microarrays 3μ slices were placed on superfrost+ glass slides, and stained and scored for ER, PR, HER2, Ki67, CK5/6, CK14, and p63. HER2 2+ tumors were evaluated using SISH (Silver In Situ Hybridization). Scoring of the IHC staining was performed by a breast pathologist (VJ). A 10% cut-off was used to define a positive staining for all markers, except Ki67: low if ≤14% and high if >14% according to the St Gallen guidelines of 2013 [35], and HER2: negative if 0/1+ and positive if 2+(SISH confirmed)/3+. Tumors were defined as basal-like if at least one out of three basal markers (CK14, CK5/6, P63) were positive. We also classified the tumors using the St Gallen guidelines of 2013 using ER, PR, HER2, and Ki67 [35,36].

### Diabetes treatment classification

Diabetes status was determined based on medical diagnosis from the National Patient Register. Diabetes duration was defined as time from age of diabetes diagnosis till age of breast cancer diagnosis. Women with diabetes were classified as insulin users if at least 2 prescriptions of insulin were prescribed cumulatively in the period up to one year prior to breast cancer diagnosis. Exposure time was defined as time from age of start of insulin till age of breast cancer diagnosis. For women treated with other non-insulin antidiabetic drugs, the same method was used. Women with diabetes treated with insulin only were considered patients with type 1 diabetes, if they had a recorded diagnosis of type 1 diabetes (n = 21), or a medical code was missing but they were under age 30 years at diabetes diagnosis (n = 4). All other women with diabetes were considered type 2.

### Imputation

For women with unknown menopausal status (n = 5), age over 52 years [37] was used as a proxy for postmenopausal status. Missing values for BMI (n = 51 in women with diabetes, n = 42 in women without diabetes) were imputed using Multivariate Imputations by Chained Equations [38] in R studio with a predictive mean matching regression model for each analyzed dataset, imputing variables with ascending number of missing values; number of imputations = 10, number of iterations = 25; see (S1 Table). We assumed that data was missing at random and could be imputed because of correlations with other variables (S2 and S3 Tables). Variables derived from the DBCG, i.e., age of breast cancer diagnosis, year of breast cancer diagnosis, menopausal status (for analyses in all women), breast cancer treatment; the electronic patient files, i.e., smoking, alcohol, height, weight, HbA_1_C levels; the National Patient Registry, i.e., diabetes type, diabetes duration, cardiovascular disease, microvascular disease, income, education; the Danish Register of Medicinal Products Statistics, i.e., diabetes medication, hormone replacement treatment and oral contraception use; and data on breast cancer characteristics and clinicopathological subtypes. In the subsequent analyses, we only included the variables relevant for the prediction of clinicopathological subtype, i.e. age, menopausal status, smoking, alcohol, BMI, HbA_1_C, diabetes duration, oral contraception use and hormone replacement treatment.

### Statistical analyses

Patient and breast cancer characteristics at diagnosis were compared between breast cancer patients with and without diabetes using chi-square tests. Multivariable logistic regression models were used to estimate the association between diabetes status or insulin treatment with primary breast cancer clinicopathological subtypes. We constructed separate logistic regression models for each exposure (diabetes or insulin) to evaluate tumor subtype (various definitions) as model-specific outcomes. Multinomial logistic regression models were used for tumor subtypes which consisted of >2 categories. We tested for heterogeneity between insulin and non-insulin users in analysis restricted to diabetes patients only. In the analyses comparing women with and without diabetes, potential covariates were added in a one by one-stepwise manner; however, none of the covariates changed the beta-estimate for diabetes with >10% for any of the subtype classifications, except for BMI in the analysis of PR status and ER-/PR- in premenopausal women. Nonetheless, we are also showing adjusted models with breast cancer subtypes for age and BMI, because previous literature has shown associations between age, BMI and breast cancer subtypes [31]. Models for grade were adjusted for age only.

Modifications of the associations between diabetes status and breast cancer subtypes by menopausal status, BMI, and diabetes type were assessed using interactions terms. Although we found no statistically significant interactions between menopausal status and diabetes status (the lowest p-value was 0.07 in the analyses of PR), we show results for pre- and postmenopausal women separately based on previous evidence for different risk profiles [31]. To exclude potential bias by the inclusions of women with type 1 diabetes we performed a sensitivity analysis excluding women with type 1 diabetes. Moreover, explorative analyses were performed within women with type 1 and type 2 diabetes. SAS Enterprise guide 4.2 for Windows was used for statistical analyses.

## Results

This cross-sectional study consisted of 211 women with diabetes and 101 women without diabetes, all diagnosed with breast cancer and with tumor tissue available (Fig 1). Breast cancer patients with diabetes had a similar distribution of menopausal status (as a result of the age-stratified selection), but were more often obese (BMI ≥30) (p <0.0001), compared to those without diabetes (Table 1). The majority of women with diabetes (88.2%) were diagnosed with type 2 diabetes and the mean diabetes duration was 8.9 years (S3 Table). Twenty-five percent (n = 53) of the women with diabetes were treated with insulin; including 18 combined with non-insulin antidiabetic drugs. The non-insulin users were treated with non-insulin antidiabetic drugs (35%) or diabetes was controlled by diet and exercise only (40%) (S3 Table). The mean duration of insulin use was 8.4 years (S3 Table). Insulin users (47% type 1 diabetes women) were more often premenopausal compared to non-insulin users (p = 0.04); and insulin users with premenopausal breast cancer had lower BMI compared to those not treated with insulin (p = 0.0003) (S4 Table).

**Table 1:** Characteristics of breast cancer patients with and without diabetes.

	Women with breast cancer		
	Diabetes (n = 211)	No Diabetes (n = 101)	P ^d^
**Age**, median (IQ range) ^a^^,^ ^b^			
≤ 50 years	47.0 (43.0–50.0)	47.0 (43.0–50.0)	
> 50 years	67.0 (60.0–75.0)	67.0 (62.0–73.0)	
	**% (n)**	**% (n)**	
**Year of breast cancer diagnoses** ^a^			
2000–2002	12.8 (27)	6.9 (7)	
2003–2004	15.6 (33)	16.8 (17)	
2005–2006	17.5 (37)	33.7 (34)	
2007–2008	27.5 (58)	18.8 (19)	
2009–2010	26.6 (56)	23.8 (24)	
**Menopausal status** ^b^			0.57
Pre	52.1 (110)	48.5 (49)	
Post	47.9 (101)	51.5 (52)	
**BMI in kg/m**^**2**^ ^c^			
Premenopausal women			0.0002
<25 (normal)	30.3 (27)	46.7 (14)	
≥25 (overweight)	24.7 (22)	50.0 (15)	
≥30 (obese)	44.9 (40)	<5 (<5) ^ǂ^	
Postmenopausal women			0.005
<25 (normal)	22.5 (16)	55.2 (16)	
≥25 (overweight)	38.0 (27)	31.0 (9)	
≥30 (obese)	39.4 (28)	<14 (<5) ^ǂ^	
**Morphology**			0.54
Ductal	75.8 (160)	70.3 (71)	
Lobular	7.6 (16)	10.9 (11)	
Other	16.6 (35)	18.8 (19)	
**Tumour size in mm**			
≤ 20	57.8 (122)	57.4 (58)	0.54
21–50	36.5 (77)	39.6 (40)	
>50	5.7 (12)	<5 (<5) ^ǂ^	
**Number of positive lymph nodes**			0.50
0	50.3 (102)	54.0 (54)	
1–3	32.5 (66)	26.0 (26)	
>3	17.2 (35)	20.0 (20)	
**Grade**			0.03
Grade 1	20.3 (41)	19.0 (19)	
Grade 2	35.6 (72)	51.0 (51)	
Grade 3	44.1 (89)	30.0 (30)	
**ER**			0.08
Positive	77.6 (163)	86.1 (87)	
Negative	22.4 (47)	13.9 (14)	
**PR**			0.17
Positive	64.4 (136)	72.3 (73)	
Negative	35.6 (75)	27.7 (28)	
**HER2**			0.07
Positive	10.5 (22)	17.8 (18)	
Negative	89.5 (187)	82.2 (83)	

^a^ Matching variable,

^b^ at breast cancer diagnosis,

^c^ closest measure prior to breast cancer diagnosis,

^d^ Chi-square test. Missing values are not shown, therefore the sum of the categories does not add up to the total number of patients for BMI, positive lymph nodes, grade, ER and HER2.

^ǂ^ Exact numbers <5 with percentages cannot be shown according to regulations of Statistics Denmark.

*IQ = interquartile range*, *BMI = Body Mass Index*.

### Association between diabetes and clinicopathological breast cancer subtypes

Breast cancer patients with diabetes had a similar distribution of morphology, tumor size, and number of positive lymph nodes compared to those without diabetes (Table 1); also if stratified for menopausal status (S5 Table).

Premenopausal breast cancer patients with diabetes had more often PR-negative (OR = 2.44(95%CI:1.07–5.55), p = 0.03), HER2-negative (OR = 2.84(95%CI:1.11–7.21), p = 0.03), and basal-like (OR = 3.14(95%CI:1.03–9.60), p = 0.05) tumors than those without diabetes, with non-statically significant increased frequencies of ER-negative (OR = 2.48(95%CI:0.95–6.45)) and triple negative (OR = 2.60(95%CI:0.88–7.67) tumors (Table 2 and S6 Table). After adjustment for age and BMI, the associations remained similar in size but less statistically significant. We found no statistically significant associations between diabetes status and grade or Ki67, nor using the more refined St. Gallen subtyping (Table 2 and S6 Table). We found no modification of breast cancer subtype by BMI or diabetes type. Sensitivity analyses, in which women with type 1 diabetes were excluded, resulted in hazard ratios of the same direction and similar size (S7 Table). We did not find an association between any of the clinicopathological breast cancer subtypes and diabetes in postmenopausal women (Table 2). In analyses including all women, we only found statistically significant more basal-like tumors in women with diabetes compared to those without (OR = 2.39(95%CI:1.07–5.35), p = 0.03).

**Table 2:** Crude and adjusted odds ratios for breast cancer clinicopathological subtypes of women with diabetes compared to women without diabetes in subgroups of menopausal status using (multinomial) logistic regression.

**Premenopausal women with breast cancer**				
**Dependent variable**	**Independent variable of exposure**			
	**Diabetes vs. No Diabetes**		**Diabetes vs. No Diabetes**	
	**crude OR (95% CI)**	**P**	**adjusted OR** * **(95% CI)**	**P**
Grade 2 (vs. grade 1)	0.56 (0.22–1.42)	0.22	0.56 (0.22–1.42)	0.22
Grade 3 (vs. grade 1)	1.02 (0.40–2.61)	0.97	1.08 (0.41–2.86)	0.88
ER- (vs. ER+)	2.48 (0.95–6.45)	0.06	2.32 (0.86–6.31)	0.10
PR- (vs. PR+)	**2.44 (1.07–5.55)**	**0.03**	2.18 (0.92–5.17)	0.07
HER2- (vs. HER2+)	**2.84 (1.11–7.22)**	**0.03**	**2.94 (1.08–8.02)**	**0.04**
High ki67 (vs. low ki67)	1.23 (0.62–2.42)	0.55	1.17 (0.53–2.58)	0.70
Basal-like ^a^ (vs. non-basal-like)	**3.14 (1.03–9.60)**	**0.05**	**3.11 (0.98–9.86)**	**0.05**
ER+/PR- (vs. ER+/PR+)	2.10 (0.55–7.96)	0.28	1.77 (0.43–7.18)	0.42
ER-/PR- (vs. ER+/PR+)	**2.67 (1.02–7.00)**	**0.05**	2.46 (0.90–6.75)	0.08
Luminal B-like, HER2- ^c^ (vs. luminal A-like ^b^)	1.15 (0.47–2.82)	0.76	1.05 (0.40–2.73)	0.92
HER2+ ^d^ (vs. luminal A-like)	0.46 (0.17–1.23)	0.12	0.41 (0.14–1.20)	0.10
Triple-negative ^e^ (vs. luminal A-like)	2.60 (0.88–7.67)	0.08	2.21 (0.71–6.69)	0.17
**Postmenopausal women with breast cancer**				
**Dependent variable**	**Independent variable of exposure**			
	**Diabetes vs. No Diabetes**		**Diabetes vs. No Diabetes**	
	**crude OR (95% CI)**	**P**	**adjusted OR** * **(95% CI)**	**P**
Grade 2 (vs. grade 1)	0.80 (0.32–2.04)	0.65	0.80 (0.31–2.03)	0.64
Grade 3 (vs. grade 1)	1.97 (0.72–5.39)	0.19	1.97 (0.72–5.39)	0.19
ER- (vs. ER+)	1.27 (0.52–3.14)	0.60	1.33 (0.52–3.40)	0.55
PR- (vs. PR+)	0.96 (0.48–1.93)	0.92	1.06 (0.51–2.19)	0.88
HER2- (vs. HER2+)	1.15 (0.43–3.13)	0.78	1.20 (0.40–3.59)	0.75
High ki67 (vs. low ki67)	1.11 (0.56–2.22)	0.77	1.06 (0.52–2.18)	0.87
Basal-like ^a^ (vs. non-basal-like)	1.62 (0.50–5.29)	0.43	1.73 (0.51–5.91)	0.38
ER+/PR- (vs. ER+/PR+)	0.79 (0.33–1.87)	0.59	0.89 (0.36–2.19)	0.79
ER-/PR- (vs. ER+/PR+)	1.20 (0.48–3.04)	0.69	1.29 (0.49–3.39)	0.60
Luminal B-like, HER2- ^c^ (vs. luminal A-like ^b^)	0.65 (0.29–1.44)	0.29	0.58 (0.25–1.35)	0.21
HER2+ ^d^ (vs. luminal A-like)	0.79 (0.28–2.26)	0.66	0.88 (0.28–2.71)	0.82
Triple-negative ^e^ (vs. luminal A-like)	1.29 (0.41–4.00)	0.66	1.30 (0.40–4.20)	0.67

Logistic regression for tumor subtypes with 2 categories and multinomial logistic regression for tumor subtype with >2 categories as the dependent variable.

^a^ Positive for ≥1 of the basal markers CK56, CK14, and P63,

^b^ ER+, PR+, HER2-, low Ki67,

^c^ ER+, PR-, HER2- with high Ki67,

^d^ ER+ or ER-, PR+ or PR-, HER2+,

^e^ ER-, PR-, HER2-.

* Adjusted for age and BMI (continuous), except for grade, which is adjusted for age only.

*OR = Odds Ratio*, *CI = Confidence Interval*.

### Association between insulin treatment and clinicopathological breast cancer subtypes

Tumor morphology, tumor size and number of positive lymph nodes did not differ between women with diabetes treated with or without insulin (S4 Table); similar results were found in analyses stratified for menopausal status (data not shown).

We observed no statistically significant evidence for the development of poor prognosis tumors among insulin users (Table 3 and S8 Table). Premenopausal women with diabetes not using insulin were more likely to develop ER-negative (OR = 3.06(95%CI:1.30–7.20), p = 0.01) and PR-negative (OR = 2.98(95%CI:1.11–8.00), p = 0.03) compared to women without diabetes, while ORs for ER and PR-negative tumors in insulin users compared to women without diabetes were only slightly increased (Table 3 and S8 Table). We performed explorative analyses separately in type 1 and type 2 insulin-treated premenopausal women with diabetes trying to understand these differences between insulin and non-insulin users. The associations between diabetes and tumor subtypes among type 1 diabetes insulin users were more in line with the findings in the non-insulin users (e.g. poor prognosis tumors), while we observed a suggestion that type 2 diabetes insulin users had better prognosis tumors (S8 and S9 Tables). However, overall, there was no evidence for a statistically significant heterogeneity between insulin and non-insulin users for any of the clinicopathological subtypes in the analyses restricted to breast cancer patients with diabetes (Table 3). In addition, adjustment for age and BMI did not materially change the effect estimates or their 95% confidence intervals (S8 and S10 Tables). In postmenopausal women, we observed no association of insulin, with breast cancer subtypes (Table 3). We did not have enough power to include subtypes using the more refined St Gallen criteria in the analyses stratified by menopausal status. In analyses including all women, we found significantly more basal-like tumors (OR = 2.5(95%CI:1.09–5.74), p = 0.03) and ER-/PR-negative tumors (OR = 1.99(95%CI:1.00–3.95), p = 0.05) in non-insulin users compared to women without diabetes.

**Table 3:** Crude and adjusted odds ratios for breast cancer clinicopathological subtypes of women with diabetes treated with or without insulin compared to women without diabetes in subgroups of menopausal status using (multinomial) logistic regression.

**Premenopausal women with breast cancer**					
**Dependent variable**	**Independent variable of exposure**				
	**Insulin** * **vs. No Diabetes**		**No Insulin** ^†^ **vs. No Diabetes**		**Diabetes only**
					**Insulin vs. No Insulin**
	**crude OR (95% CI)**	**P**	**crude OR (95% CI)**	**P**	**P**
Grade 2 (vs. grade 1)	0.55 (0.18–1.68)	0.29	0.57 (0.21–1.58)	0.28	0.93
Grade 3 (vs. grade 1)	0.53 (0.16–1.74)	0.30	1.34 (0.49–3.67)	0.57	0.09
ER- (vs. ER+)	1.54 (0.45–5.24)	0.49	**2.98 (1.11–8.00)**	**0.03**	0.20
PR- (vs. ER+)	1.37 (0.47–4.00)	0.57	**3.06 (1.30–7.20)**	**0.01**	0.08
HER2- (vs. ER+)	**8.97 (1.10–73.36)**	**0.04**	2.16 (0.82–5.67)	0.12	0.19
High ki67 (vs. low ki67)	0.80 (0.32–1.96)	0.62	1.48 (0.72–3.05)	0.29	0.15
**Postmenopausal women with breast cancer**					
**Dependent variable**	**Independent variable of exposure**				
	**Insulin** * **vs. No Diabetes**		**No Insulin** ^†^ **vs. No Diabetes**		**Diabetes only**
					**Insulin vs. No Insulin**
	**crude OR (95% CI)**	**P**	**crude OR (95% CI)**	**P**	**P**
Grade 2 (vs. grade 1)	0.60 (0.12–2.96)	0.53	0.85 (0.32–2.25)	0.75	0.66
Grade 3 (vs. grade 1)	2.05 (0.43–9.78)	0.37	1.95 (0.69–5.55)	0.21	0.95
ER- (vs. ER+)	1.47 (0.39–5.58)	0.57	1.23 (0.38–3.15)	0.66	0.78
PR- (vs. ER+)	1.01 (0.34–3.01)	0.98	0.95 (0.46–1.96)	0.89	0.90
HER2- (vs. ER+)	0.83 (0.19–3.60)	0.80	1.26 (0.44–3.63)	0.67	0.56
High ki67 (vs. low ki67)	0.80 (0.26–2.46)	0.70	1.19 (0.58–2.45)	0.63	0.46

Logistic regression for tumor subtypes with 2 categories and multinomial logistic regression for tumor subtype with >2 categories as the dependent variable.

* Women with diabetes treated with insulin (analogues) regardless the use of concomitant non-insulin antidiabetic drugs,

^†^ women with diabetes treated only with diet and exercise and users of non-insulin antidiabetic drugs only.

*OR = Odds Ratio*, *CI = Confidence Interval*.

## Discussion

We found no compelling evidence that women with diabetes develop different clinicopathological subtypes compared to women without diabetes. However, premenopausal breast cancer patients with diabetes tend to develop breast tumors that do not express hormonal receptors and basal-like tumors, which are typically associated with poor prognosis. The majority of the women in our population had type 2 diabetes mellitus, so the results are most applicable for these patients. We also found no strong evidence that insulin treatment is associated with clinicopathological subtypes; though the poor-prognosis tumors were more often occurring in premenopausal women with diabetes *not* using insulin and in type 1 diabetes insulin users.

Only a few studies have investigated breast cancer characteristics among women with diabetes [20,22,39,40]. Two previous studies stratified the results for menopausal status and they also found that premenopausal women developed more often tumors that were hormone receptor negative [22,39], after multivariable adjustment [39]. Overall results were consistent with ours, showing more ER-negative, PR-negative and HER2-negative tumors in women with diabetes, with relative frequencies of 1.5 to 2.5, but most differences were not statistically significant, except for PR [20,22] and ER, even after adjustment for BMI [40]. A few studies that reported tumor markers (ER, PR, and some HER2 status) among women with diabetes [11–13,26,41] compared (breast cancer) mortality or disease-free survival among women with and without diabetes as their primary objective. Therefore, only crude estimates of associations between diabetes and tumor subtype were reported and not stratified for menopausal status. Women included in these studies were mainly postmenopausal and no significant associations were found between tumor markers and diabetes status.

Studies on the association between diabetes treatment and breast cancer subtype are even more scarce. No difference in tumor stage and tumor subtype among glargine versus non-glargine users was previously described [42,43]. Studies that compared metformin users to women with diabetes treated with sulphonylurea or insulin (non-metformin) showed no difference in ER status [20,44], but sulphonylurea or insulin users presented more PR-negative tumors (63.0% versus 26.7%, p = 0.041) [44] and more HER-2 positive (29.5% versus 21%, p = 0.002) [20] than in the metformin-treated subgroup.

Our study was based on the comprehensive biobanks (archival tumor tissue from a randomly selected group of women), and databases available in Denmark, and included medication history at least five years prior to breast cancer diagnosis from prescription records, resulting in a patient selection minimally affected by survival, selection or ascertainment bias. Due to oversampling of young breast cancer patients, we could examine the association between diabetes and clinicopathological subtypes in both pre- and post-menopausal women. An experienced breast pathologist reviewed all tumor samples and we had complete data on IHC markers (including basal markers). All IHC stainings were validated and performed in one center and scored by the same breast pathologist, to prevent inter-laboratory and inter-observer variability [45,46] and to assure quality of the data. Additionally, data on risk factors such as BMI were obtained and effect estimates were adjusted for potential confounders and we followed the STROBE recommendations in reporting scientific research (S11 Table).

Our study was only sufficiently powered (around 80%; likelihood-ratio test with a two-sided p-value of 0.05) to detect large differences between breast cancer subtypes, e.g. 80% versus 60% ER-positive tumors, in women with and without diabetes and therefore, subtle differences may not have been detected. Furthermore, given the design of our study, in which odds ratios may represent on overestimation of the real risks, validation using prospective cohort analyses is recommended. Unfortunately, we had insufficient power for separate analyses of diabetes type 1 and different insulin analogues. We had also limited power to investigate the duration/dose of insulin exposure and the effect on breast cancer subtype. However, the majority of insulin users had prescriptions of insulin over several years.

We had no information on whether breast cancer patients were mammography screen-detected or not. Breast cancer subtype of screen-detected tumors differs from tumors found outside of screening [47] and there may be a higher non-participation for screening among postmenopausal women with diabetes compared to women without diabetes [48]. However, Danish national screening programs started only in 2007 for women aged 50–69 [49]. All statistically significant differences in our study were found in premenopausal women <52 years, which were mostly not screened, because the use of opportunistic screening in Denmark is low [49].

BMI, HbA_1_C and other risk factors such as alcohol and smoking were collected from the medical records of patients and were incomplete. However, since we had extensive data on variables associated with e.g. BMI, we were able to impute missing values using multiple imputations. Although the ratios for observed and imputed BMI were similar, BMI could still be misclassified for some patients. However, we think that misclassification of BMI is unlikely to influence our results, since BMI did not affect the association between diabetes and breast cancer subtype, except for PR status in premenopausal women. Nevertheless, we have to interpret both our positive and null results with caution.

There may be several reasons why we found a stronger and significant association between hormone receptor negative tumors and diabetes in premenopausal compared to postmenopausal women. Differences in levels of BMI-related and reproductive hormones, i.e., factors related to menopausal status, such as insulin, estrogen and adipokine, may play a role in tumor subtype formation. However, in contrast to what we have observed in postmenopausal women, a previous study showed increased estrogen levels in women with diabetes [19], which would imply that postmenopausal women would more often develop ER-positive tumors.

For the interpretation of the results, it is important to realize that diabetes and BMI are strongly associated. Women with diabetes are more likely to be obese, and premenopausal obese women tend to develop hormone receptor negative tumors [50]. Such an association between BMI and hormone receptor negative breast cancer has not been observed in postmenopausal women. Our results on the association between diabetes and breast cancer subtypes are in line with these findings, even after adjustment for BMI. The same has been reported by two other studies [39,40], which might indicate that diabetes itself contributes to higher rates of hormone receptor negative breast cancer in obese women. Our observation that poor prognosis tumors are unlikely to occur more often in premenopausal women using insulin, is in line with the earlier reports that insulin (analogues) do not increase the risk of breast cancer overall [10]. However, more research is needed for type 1 diabetes.

Diabetes medication depends on the type of diabetes, as well as the severity (insulin dependent, no endogenous insulin versus insulin resistant, high levels of endogenous insulin) and duration of diabetes. Not much is known about the mechanism, by which insulin treatment would possibly influence the receptor phenotype of breast cancer. It has been shown that insulin can induce ER and PR expression, which leads to increased binding capacity of ER in MCF-7 breast cancer cell line [51]. This may suggest that women with diabetes treated with insulin would develop more ER and PR-positive tumors, which we did not observe. Moreover, the interpretation and translation of *in vivo* and *in vitro* studies to the human setting is difficult [5].

In summary, our findings suggest that premenopausal women with diabetes tend to develop triple negative and basal tumors, which are typically associated with poor prognosis. Though our study had limited power, our results warrant further investigation and future studies should stratify their analyses by menopausal status.

## Supporting Information

S1 Table Average Body Mass Index of breast cancer patients in subgroups of menopausal status, in the ten imputed datasets (% (n)). (DOCX)

S2 Table Characteristics of breast cancer patients with and without diabetes used for imputation of Body Mass Index. (DOCX)

S3 Table Patient characteristics and medication use among women with type 1 and type 2 diabetes *. (DOCX)

S4 Table Characteristics of breast cancer patients with diabetes treated with and without insulin. (DOCX)

S5 Table Tumor characteristics of breast cancer patients with and without diabetes in subgroups of menopausal status. (DOCX)

S6 Table Numbers and proportions of breast cancer clinicopathological subtypes of breast cancer patients with diabetes, type 2 diabetes and without diabetes in subgroups of menopausal status. (DOCX)

S7 Table Crude and adjusted odds ratios for breast cancer clinicopathological subtypes of women with type 2 diabetes compared to women without diabetes in subgroups of menopausal status using (multinomial) logistic regression. (DOCX)

S8 Table Number and proportion of breast cancer clinicopathological subtypes of breast cancer patients with diabetes (type 1 and type 2) treated with or without insulin in subgroups of menopausal status. (DOCX)

S9 Table Crude odds ratios for breast cancer clinicopathological subtypes of premenopausal women with type 1 or type 2 diabetes treated with insulin compared to women without diabetes using (multinomial) logistic regression. (DOCX)

S10 Table Adjusted odds ratios for breast cancer clinicopathological subtypes of women with diabetes treated with or without insulin compared to women without diabetes in subgroups of menopausal status using (multinomial) logistic regression. (DOCX)

S11 Table STROBE statement. (DOC)
