# Supplementary material for: Diabetes and Breast Cancer Subtypes
Source: PLoS One. 2017 Jan 11;12(1):e0170084. doi: 10.1371/journal.pone.0170084 (PMC5226802; doi:10.1371/journal.pone.0170084)
Supplement: S1 Table — (DOCX) [file pone.0170084.s001.docx]

**S1 Table. Average Body Mass Index of breast cancer patients in subgroups of menopausal status, in the ten imputed datasets (% (n)).**

|  | **Premenopausal women with breast cancer** | | |
| --- | --- | --- | --- |
|  | **Diabetes** (n=110^x^10) |  | **No Diabetes** (n=49^x^10) |
| **BMI in kg/m^2 a^** |  |  |  |
| <25 (normal) | 33.0 (363) |  | 48.6 (238) |
| ≥25 (overweight) | 27.3 (300) |  | 43.9 (215) |
| ≥30 (obese) | 39.7 (437) |  | 7.6 (37) |
|  |  | | |
|  | **Postmenopausal women with breast cancer** | | |
|  | **Diabetes** (N=101^x^10) |  | **No Diabetes** (N=52^x^10) |
| **BMI in kg/m^2 a^** |  |  |  |
| <25 (normal) | 26.1 (264) |  | 49.2 (256) |
| ≥25 (overweight) | 36.7 (371) |  | 32.1 (167) |
| ≥30 (obese) | 37.1 (375) |  | 18.7 (97) |

^a^ Closest measure prior to breast cancer diagnosis. Imputation was done separately for pre- and postmenopausal women. *BMI=Body Mass Index.*
