# Supplementary material for: Diabetes and Breast Cancer Subtypes
Source: PLoS One. 2017 Jan 11;12(1):e0170084. doi: 10.1371/journal.pone.0170084 (PMC5226802; doi:10.1371/journal.pone.0170084)
Supplement: S2 Table — (DOCX) [file pone.0170084.s002.docx]

**S2 Table.** **Characteristics of breast cancer patients with and without diabetes used for imputation of Body Mass Index.**

|  | **Women with breast cancer** | | |
| --- | --- | --- | --- |
|  | **Diabetes** (n=211) |  | **No Diabetes** (n=101) |
| **Height,** mean ± SD | 165.4 ± 6.2 |  | 165.4 ± 6.4 |
| **Weight,** median (IQ range) ^a^ | 78.0 (67.0-88.0) |  | 68.0 (60.0-75.0) |
|  | **% (n)** |  | **% (n)** |
| **Smoking** ^a^ |  |  |  |
| Never | 47.4 (100) |  | 42.6 (43) |
| Former | 14.7 (31) |  | 18.8 (19) |
| Current | 20.9 (44) |  | 15.8 (16) |
| Missing | 17.1 (36) |  | 22.8 (23) |
| **Alcohol** ^a^ |  |  |  |
| No | 36.0 (76) |  | 16.8 (17) |
| Moderate, <7 glasses/week | 28.4 (60) |  | 37.6 (38) |
| Heavy, >7 glasses/week | 10.9 (23) |  | 15.8 (16) |
| Missing | 24.6 (52) |  | 29.7 (30) |
| **Income** ^b^ |  |  |  |
| Low (<200,000 Danish Krone) | 63.5 (134) |  | 45.5 (46) |
| Medium (200,000-399,999) | 32.2 (68) |  | 47.5 (48) |
| High (≥400,000) | 3.8 (8) |  | 6.9 (7) |
| Missing | 0.5 (1) |  | - |
| **Education** ^c^ |  |  |  |
| Primary | 42.6 (90) |  | 27.7 (28) |
| Secondary | 34.1 (72) |  | 38.6 (39) |
| Tertiary | 17.1 (36) |  | 30.7 (31) |
| Missing | 6.2 (13) |  | 3.0 (3) |
| **Oral contraceptive use** ^d^ |  |  |  |
| Yes | 34.6 (73) |  | 34.7 (35) |
| No | 65.4 (138) |  | 65.4 (66) |
| **Hormone replacement therapy** ^d^ |  |  |  |
| Yes | 45.5 (96) |  | 44.5 (45) |
| No | 54.5 (115) |  | 55.5 (56) |
| **Cardiovascular disease** |  |  |  |
| Yes | 19.4 (41) |  | 6.9 (7) |
| No | 80.6 (170) |  | 93.1 (94) |
| **Breast cancer treatment** |  |  |  |
| Surgery only | 15.0 (29) |  | 18.3 (17) |
| Surgery, chemotherapy + endocrine therapy | 39.9 (77) |  | 39.8 (37) |
| Surgery and endocrine therapy | 26.4 (51) |  | 28.0 (26) |
| Surgery and chemotherapy | 18.7 (36) |  | 14.0 (13) |

^a^ Closest measure prior to breast cancer diagnosis, ^b^ in year of breast cancer diagnosis, ^c^ highest attained, ^d^ at least 2 prescriptions of the drugs were prescribed cumulatively in the period up to one year prior to breast cancer diagnosis. *IQ=interquartile range, SD=standard deviation.*
