# Supplementary material for: Diabetes and Breast Cancer Subtypes
Source: PLoS One. 2017 Jan 11;12(1):e0170084. doi: 10.1371/journal.pone.0170084 (PMC5226802; doi:10.1371/journal.pone.0170084)
Supplement: S3 Table — (DOCX) [file pone.0170084.s003.docx]

**S3 Table. Patient characteristics and medication use among women with type 1 and type 2 diabetes ^*^.**

|  | **Women with breast cancer and diabetes** | | | | |
| --- | --- | --- | --- | --- | --- |
|  | **Diabetes** ^†^ (n=211) |  | **Type 1 Diabetes** (n=25) |  | **Type 2 Diabetes** (n=186) |
| **Diabetes Type**, % (n) |  |  |  |  |  |
| Type 1 | 11.8 (25) |  |  |  |  |
| Type 2 | 88.2 (186) |  |  |  |  |
| **Age diabetes diagnosis**, median (IQ range) | 46.0 (34.0-58.0) |  | 23.0 (20.0-28.0) |  | 47.5 (38.0-61.0) |
| premenopausal | 36.0 (30.0-45.0) |  | 22.0 (20.0-27.0) |  | 39.0 (32.5-45.0) |
| postmenopausal | 59.0 (52.0-69.0) |  | 29.0 (20.0-47.0) |  | 61.0 (54.0-69.0) |
| **Menopausal status**, % (n) ^a^ |  |  |  |  |  |
| Pre | 51.9 (110) |  | 76.0 (19) |  | 48.9 (91) |
| Post | 48.1 (101) |  | 24.0 (6) |  | 51.1 (95) |
| **BMI in kg/m^2^,** (%) n ^b^ |  |  |  |  |  |
| <25 (normal) | 20.4 (43) |  | 40.0 (10) |  | 17.7 (33) |
| ≥25 (overweight) | 23.2 (49) |  | 24.0 (6) |  | 23.1 (43) |
| ≥ 30 (obese) | 32.2 (68) |  | <5 (<5) ^ǂ^ |  | 36.0 (67) |
| Missing | 24.2 (51) |  | 32.0 (8) |  | 23.1 (43) |
| **Diabetes duration in years**, mean ± SD | 8.9 ± 7.7 |  | 22.3 ± 7.3 |  | 7.1 ± 5.8 |
| **Hemoglobin A1C in %,** % (n) ^c^ |  |  |  |  |  |
| Pre-diabetes and controlled; 5.7-7.9 | 14.2 (30) |  | 24.0 (6) |  | 12.9 (24) |
| Uncontrolled; ≥ 8.0 | 12.8 (27) |  | 44.0 (11) |  | 8.6 (16) |
| Missing | 73.0 (154) |  | 32.0 (8) |  | 78.5 (146) |
| **Diabetes treatment**, % (n) ^d^ |  |  |  |  |  |
| Diet and exercise | 39.8 (84) |  | - |  | 45.2 (84) |
| Non-insulin antidiabetic drugs only | 35.1 (74) |  | - |  | 39.8 (74) |
| Insulin only | 16.6 (35) |  | 100.0 (25) |  | 5.4 (10) |
| Non-insulin antidiabetic drugs and insulin | 8.5 (18) |  | - |  | 9.7 (18) |
| **Exposure time** **in years,** mean ± SD ^e^ |  |  |  |  |  |
| Any antidiabetic drugs | 6.8 ± 4.1 |  | 10.1 ± 3.5 |  | 6.0 ± 3.8 |
| Insulin | 8.4 ± 4.2 |  | 10.1 ± 3.5 |  | 6.9 ± 4.2 |
| Non-insulin antidiabetic drugs | 5.5 ± 3.6 |  | - |  | 5.5 ± 3.6 |
| **Insulin type**, % (n) |  |  |  |  |  |
| Human insulin | 23.4 (50) |  | 100.0 (25) |  | 13.4 (25) |
| Insulin analogues | 11.7 (25) |  | 44.0 (11) |  | 7.5 (14) |
| **Metformin** | 29.4 (63) |  | - |  | 33.9 (63) |

^a^ At breast cancer diagnosis, ^b^ closest measure prior to breast cancer diagnosis, ^c^ measured in varying time periods before breast cancer diagnosis, ^d^ at least 2 prescriptions of an antidiabetic drug were prescribed cumulatively in the period up to one year prior to breast cancer diagnosis, ^e^ defined as time from age of start of the antidiabetic drug till age of breast cancer diagnosis. * Used for imputation, † all women with diabetes, ^ǂ^ exact numbers <5 with percentages cannot be shown according to regulations of Statistics Denmark. *IQ=interquartile range, SD=standard deviation, BMI=Body Mass Index.*
