# Supplementary material for: Diabetes and Breast Cancer Subtypes
Source: PLoS One. 2017 Jan 11;12(1):e0170084. doi: 10.1371/journal.pone.0170084 (PMC5226802; doi:10.1371/journal.pone.0170084)
Supplement: S4 Table — (DOCX) [file pone.0170084.s004.docx]

**S4 Table. Characteristics of breast cancer patients with diabetes treated with and without insulin.**

|  | **Women with breast cancer and diabetes** | | | | |
| --- | --- | --- | --- | --- | --- |
|  | **Insulin** ^*^  (n=53) |  | **No Insulin** ^†^  (n=158) |  | **P** |
| **Age,** median (IQ range) ^a^ | 48.0 (44.0-51.0) |  | 50.0 (47.0-68.0) |  |  |
| ≤ 50 years | 47 (43.0-49.0) |  | 48.0 (44.0-50.0) |  |  |
| > 50 years | 65.0 (58.0-69.0) |  | 68.0 (61.0-75.0) |  |  |
| **Menopause** % (n) ^a^ |  |  |  |  |  |
| no | 64.2 (34) |  | 48.1 (76) |  | 0.04 |
| yes | 35.9 (19) |  | 51.9 (82) |  |  |
| **Diabetes type**  % (n) |  |  |  |  |  |
| Type 1 | 47.2 (25) |  | - |  | <0.0001 |
| Type 2 | 52.8 (28) |  | 100 (158) |  |  |
| **BMI in kg/m^2^,** median (IQ range) ^b^ |  |  |  |  |  |
| premenopausal | 24.3 (22.3-28.9) |  | 30.7 (25.9-34.3) |  |  |
| postmenopausal | 28.3 (23.2-35.0) |  | 29.1 (25.2-31.2) |  |  |
| **Morphology** % (n) |  |  |  |  | 0.49 |
| Ductal | 73.6 (39) |  | 76.6 (121) |  |  |
| Lobular | 11.3 (6) |  | 6.3 (10) |  |  |
| Others | 15.1 (8) |  | 17.1 (27) |  |  |
| **Tumor size in mm** % (n) |  |  |  |  |  |
| ≤ 20 | 62.3 (33) |  | 56.3 (89) |  | 0.73 |
| 21-50 | 32.1 (17) |  | 38.0 (60) |  |  |
| >50 | <6 (<5) ^ǂ^ |  | 5.7 (9) |  |  |
| **Number of positive lymph nodes** % (n) |  |  |  |  |  |
| 0 | 47.1 (24) |  | 51.3 (78) |  | 0.86 |
| 1-3 | 35.3 (18) |  | 31.6 (48) |  |  |
| >3 | 17.7 (9) |  | 17.1 (26) |  |  |

^a^ At breast cancer diagnosis, ^b^ closest measure prior to breast cancer diagnosis. * Women with diabetes treated with insulin (analogues) regardless the use of concomitant non-insulin antidiabetic drugs, † women with diabetes treated only with diet and exercise and users of non-insulin antidiabetic drugs only, ^ǂ^ exact numbers <5 with percentages cannot be shown according to regulations of Statistics Denmark. *IQ=interquartile range, SD=standard deviation.*
