# Supplementary material for: Diabetes and Breast Cancer Subtypes
Source: PLoS One. 2017 Jan 11;12(1):e0170084. doi: 10.1371/journal.pone.0170084 (PMC5226802; doi:10.1371/journal.pone.0170084)
Supplement: S5 Table — (DOCX) [file pone.0170084.s005.docx]

**S5 Table. Tumor characteristics of breast cancer patients with and without diabetes in subgroups of menopausal status.**

|  | **Premenopausal women with breast cancer** | | |  |  |
| --- | --- | --- | --- | --- | --- |
|  | **Diabetes** (n=110) |  | **No Diabetes** (n=49) |  | **P** ^a^ |
|  | **% (n)** |  | **% (n)** |  |  |
| **Morphology** |  |  |  |  | 0.25 |
| Ductal | 78.2 (86) |  | 71.4 (35) |  |  |
| Lobular | <5 (<5) ^ǂ^ |  | 10.2 (5) |  |  |
| Other | 18.2 (20) |  | 18.4 (9) |  |  |
| **Tumor size in mm** |  |  |  |  | 0.71 |
| ≤ 20 | 56.4 (62) |  | 63.3 (31) |  |  |
| 21-50 | 39.1 (43) |  | 32.7 (47) |  |  |
| >50 | 4.6 (5) |  | <5 (<5) ^ǂ^ |  |  |
| **Number of positive lymph nodes** |  |  |  |  | 0.57 |
| 0 | 46.4 (51) |  | 46.9 (23) |  |  |
| 1-3 | 37.3 (41) |  | 30.6 (15) |  |  |
| >3 | 16.4 (18) |  | 22.5 (11) |  |  |
|  | **Postmenopausal women with breast cancer** | | |  |  |
|  | **Diabetes** (n=101) |  | **No Diabetes** (n=52) |  | **P** ^a^ |
|  | **% (n)** |  | **% (n)** |  |  |
| **Morphology** |  |  |  |  | 0.79 |
| Ductal | 73.3 (74) |  | 69.2 (36) |  |  |
| Lobular | 11.9 (12) |  | 11.5 (6) |  |  |
| Other | 14.9 (15) |  | 19.2 (10) |  |  |
| **Tumor size in mm** |  |  |  |  | 0.18 |
| ≤ 20 | 59.4 (60) |  | 51.9 (27) |  |  |
| 21-50 | 33.7 (34) |  | 46.2 (24) |  |  |
| >50 | 6.9 (7) |  | <5 (<5) ^ǂ^ |  |  |
| **Number of positive lymph nodes** |  |  |  |  | 0.75 |
| 0 | 54.8 (51) |  | 60.8 (31) |  |  |
| 1-3 | 26.9 (25) |  | 21.6 (11) |  |  |
| >3 | 18.3 (17) |  | 17.7 (9) |  |  |

^a^ Chi-square test. Missing values are not shown, therefore the sum of the categories does not add up to the total number of patients for positive lymph nodes. ^ǂ^ Exact numbers <5 with percentages cannot be shown according to regulations of Statistics Denmark.
