# Supplementary material for: Diabetes and Breast Cancer Subtypes
Source: PLoS One. 2017 Jan 11;12(1):e0170084. doi: 10.1371/journal.pone.0170084 (PMC5226802; doi:10.1371/journal.pone.0170084)
Supplement: S6 Table — (DOCX) [file pone.0170084.s006.docx]

**S6 table. Numbers and proportions of breast cancer clinicopathological subtypes of breast cancer patients with diabetes, type 2 diabetes and without diabetes in subgroups of menopausal status.**

|  | **Premenopausal women with breast cancer** | | | | |  | **Postmenopausal women with breast cancer** | | | | |
| --- | --- | --- | --- | --- | --- | --- | --- | --- | --- | --- | --- |
|  | **Diabetes** ^*^  (n=110) |  | **Type 2 Diabetes** ^†^ (n=91) |  | **No Diabetes**  (n=49) |  | **Diabetes ^*^**  (n=101) |  | **Type 2 Diabetes** ^†^ (n=95) |  | **No Diabetes**  (n=52) |
| **Tumor subtype** | **% (n)** |  | **% (n)** |  | **% (n)** |  | **% (n)** |  | **% (n)** |  | **% (n)** |
| Grade 1 | 22.9 (25) |  | 25.6 (23) |  | 18.4 (9) |  | 17.2 (16) |  | 16.9 (15) |  | 19.6 (10) |
| Grade 2 | 33.0 (36) |  | 31.1 (28) |  | 46.9 (23) |  | 38.7 (36) |  | 39.3 (35) |  | 54.9 (28) |
| Grade 3 | 44.0 (48) |  | 43.3 (39) |  | 34.7 (17) |  | 44.1 (41) |  | 43.8 (39) |  | 25.5 (13) |
|  |  |  |  |  |  |  |  |  |  |  |  |
| ER+ | 74.3 (81) |  | 74.4 (67) |  | 87.8 (43) |  | 81.2 (82) |  | 80.0 (76) |  | 84.6 (44) |
| ER- | 25.7 (28) |  | 25.6 (23) |  | 12.2 (6) |  | 18.8 (19) |  | 20.0 (19) |  | 15.4 (8) |
| PR+ | 64.6 (71) |  | 62.6 (57) |  | 81.6 (40) |  | 64.4 (65) |  | 63.2 (60) |  | 63.5 (33) |
| PR- | 35.5 (39) |  | 37.4 (34) |  | 18.4 (9) |  | 35.6 (36) |  | 36.8 (35) |  | 36.5 (19) |
| HER2+ | 9.3 (10) |  | 10.0 (9) |  | 22.5 (11) |  | 11.9 (12) |  | 11.6 (11) |  | 13.5 (7) |
| HER2- | 90.7 (98) |  | 90.0 (81) |  | 77.6 (38) |  | 88.1 (89) |  | 88.4 (84) |  | 86.5 (45) |
| Low ki67 | 50.0 (54) |  | 50.6 (45) |  | 55.1 (27) |  | 61.0 (61) |  | 58.5 (55) |  | 63.5 (33) |
| High ki67 | 50.0 (54) |  | 49.4 (44) |  | 44.9 (22) |  | 39.0 (39) |  | 41.5 (39) |  | 36.5 (19) |
| Non-basal-like | 78.2 (86) |  | 78.0 (71) |  | 91.8 (45) |  | 88.1 (89) |  | 88.4 (84) |  | 92.3 (48) |
| Basal-like ^a^ | 21.8 (24) |  | 22.0 (20) |  | <9 (<5) ^ǂ^ |  | 11.9 (12) |  | 11.6 (11) |  | <9 (<5) ^ǂ^ |
|  |  |  |  |  |  |  |  |  |  |  |  |
| ER+/PR+ | 64.2 (70) |  | 62.2 (56) |  | 81.6 (40) |  | 64.4 (65) |  | 20.0 (19) |  | 63.5 (33) |
| ER+/PR- | 10.1 (11) |  | 12.2 (11) |  | <7 (<5) ^ǂ^ |  | 16.8 (17) |  | 16.8 (16) |  | 21.2 (11) |
| ER-/PR- | 25.7 (28) |  | 25.6 (23) |  | 12.2 (6) |  | 18.8 (19) |  | 63.2 (60) |  | 15.4 (8) |
|  |  |  |  |  |  |  |  |  |  |  |  |
| Luminal A-like ^b^ | 43.8 (46) |  | 43.7 (38) |  | 46.9 (23) |  | 50.0 (50) |  | 47.9 (45) |  | 44.2 (23) |
| Luminal B-like, HER2- ^c^ | 21.9 (23) |  | 21.8 (19) |  | 20.4 (10) |  | 24.0 (24) |  | 25.5 (24) |  | 32.7 (17) |
| HER2+ ^d^ | 9.5 (10) |  | 10.3 (9) |  | 22.5 (11) |  | 12.0 (12) |  | 11.7 (11) |  | 13.5 (7) |
| Triple-negative ^e^ | 24.8 (26) |  | 24.1 (21) |  | 10.2 (5) |  | 14.0 (14) |  | 14.9 (14) |  | 9.6 (5) |

^a^ Positive for ≥1 of the basal markers CK56, CK14, and P63, ^b^ ER+, PR+, HER2-, low Ki67, ^c^ ER+, PR-, HER2- with high Ki67, ^d^  ER+ or ER-, PR+ or PR-, HER2+, ^e^ ER-, PR-, HER2-. Missing values are not shown, therefore the sum of the categories does not add up to the total number of patients. * All women with diabetes, † women with type 2 diabetes only, ^ǂ^ exact numbers <5 with percentages cannot be shown according to regulations of Statistics Denmark.
