# Supplementary material for: Diabetes and Breast Cancer Subtypes
Source: PLoS One. 2017 Jan 11;12(1):e0170084. doi: 10.1371/journal.pone.0170084 (PMC5226802; doi:10.1371/journal.pone.0170084)
Supplement: S7 Table — (DOCX) [file pone.0170084.s007.docx]

**S7 Table. Crude and adjusted odds ratios for** **breast cancer clinicopathological subtypes of women with type 2 diabetes compared to women without diabetes in subgroups of menopausal status using (multinomial) logistic regression.**

| **Premenopausal women with breast cancer** | | | | | |
| --- | --- | --- | --- | --- | --- |
|  | **Independent variable of exposure** | | | | |
|  | **Type 2 Diabetes vs. No Diabetes** | |  | **Type 2 Diabetes vs. No Diabetes** | |
| **Dependent variable** | **crude OR (95% CI)** | **P** |  | **adjusted OR ^*^ (95% CI)** | **P** |
| Grade 2 (vs. grade 1) | 0.48 (0.19-1.23) | 0.13 |  | **0.34 (0.12-0.96)** | **0.04** |
| Grade 3 (vs. grade 1) | 0.90 (0.34-2.34) | 0.83 |  | 0.66 (0.21-2.00) | 0.46 |
|  |  |  |  |  |  |
| ER- (vs. ER+) | 2.46 (0.93-6.53) | 0.07 |  | 2.36 (0.82-6.83) | 0.11 |
| PR- (vs. PR+) | **2.65 (1.15-6.13)** | **0.02** |  | **2.50 (1.00-6.26)** | **0.05** |
| HER2- (vs. HER2+) | **2.61 (1.00-6.82)** | **0.05** |  | 2.62 (0.90-7.62) | 0.08 |
| High ki67 (vs. low ki67) | 1.20 (0.60-2.42) | 0.61 |  | 1.17 (0.53-2.58) | 0.70 |
| Basal-like ^a^ (vs. non-basal-like) | **3.17 (1.02-9.87)** | **0.05** |  | 3.12 (0.93-10.48) | 0.07 |
|  |  |  |  |  |  |
| ER+/PR- (vs. ER+/PR+) | 2.62 (0.69-10.00) | 0.16 |  | 2.49 (0.59-10.44) | 0.21 |
| ER-/PR- (vs. ER+/PR+) | **2.74 (1.02-7.34)** | **0.04** |  | 2.67 (0.91-7.86) | 0.08 |
|  |  |  |  |  |  |
| Luminal B-like, HER2- ^c^ (vs. luminal A-like ^b^) | 1.15 (0.46-2.90) | 0.77 |  | 1.04 (0.38-2.86) | 0.94 |
| HER2+ ^d^ (vs. luminal A-like) | 0.50 (0.18-1.38) | 0.18 |  | 0.46 (0.15-1.43) | 0.18 |
| Triple-negative ^e^ (vs. luminal A-like) | 2.54 (0.83-7.67) | 0.10 |  | 2.05 (0.61-6.85) | 0.25 |

| **Postmenopausal women with breast cancer** | | | | | |
| --- | --- | --- | --- | --- | --- |
|  | **Independent variable of exposure** | | | | |
|  | **Type 2 Diabetes vs. No Diabetes** | |  | **Type 2 Diabetes vs. No Diabetes** | |
| **Dependent variable** | **crude OR (95% CI)** | **P** |  | **adjusted OR ^*^ (95% CI)** | **P** |
| Grade 2 (vs. grade 1) | 0.83 (0.33-2.14) | 0.70 |  | 0.88 (0.33-2.35) | 0.80 |
| Grade 3 (vs. grade 1) | 2.00 (0.72-5.53) | 0.18 |  | 1.88 (0.65-5.39) | 0.24 |
|  |  |  |  |  |  |
| ER- (vs. ER+) | 1.38 (0.56-3.40) | 0.49 |  | 1.43 (0.56-3.67) | 0.46 |
| PR- (vs. PR+) | 1.01 (0.50-2.04) | 0.97 |  | 1.09 (0.52-2.28) | 0.81 |
| HER2- (vs. HER2+) | 1.19 (0.43-3.28) | 0.74 |  | 1.04 (0.35-3.06) | 0.96 |
| High ki67 (vs. low ki67) | 1.23 (0.61-2.48) | 0.56 |  | 1.18 (0.57-2.44) | 0.66 |
| Basal-like ^a^ (vs. non-basal-like) | 1.57 (0.48-5.21) | 0.46 |  | 1.70 (0.49-5.88) | 0.40 |
|  |  |  |  |  |  |
| ER+/PR- (vs. ER+/PR+) | 0.80 (0.33-1.92) | 0.62 |  | 0.88 (0.35-2.19) | 0.78 |
| ER-/PR- (vs. ER+/PR+) | 1.31 (0.52-3.31) | 0.57 |  | 1.39 (0.53-3.65) | 0.51 |
|  |  |  |  |  |  |
| Luminal B-like, HER2- ^c^ (vs. luminal A-like ^b^) | 0.72 (0.33-1.60) | 0.42 |  | 0.64 (0.27-1.50) | 0.30 |
| HER2+ ^d^ (vs. luminal A-like) | 0.80 (0.46-4.46) | 0.69 |  | 0.89 (0.28-2.80) | 0.85 |
| Triple-negative ^e^ (vs. luminal A-like) | 1.43 (0.46-4.46) | 0.54 |  | 1.42 (0.44-4.61) | 0.56 |

Logistic regression for tumor subtypes with 2 categories and multinomial logistic regression for tumor subtype with >2 categories as the dependent variable. Sensitivity analyses excluding women with type 1 diabetes. ^a^ Positive for ≥1 of the basal markers CK56, CK14, and P63, ^b^ ER+, PR+, HER2-, low Ki67, ^c^ ER+, PR-, HER2- with high Ki67, ^d^  ER+ or ER-, PR+ or PR-, HER2+, ^e^ ER-, PR-, HER2-. * Adjusted for age and BMI (continuous), except for grade which is adjusted for age only. *OR=Odds Ratio, CI=Confidence Interval.*
