# Supplementary material for: Diabetes and Breast Cancer Subtypes
Source: PLoS One. 2017 Jan 11;12(1):e0170084. doi: 10.1371/journal.pone.0170084 (PMC5226802; doi:10.1371/journal.pone.0170084)
Supplement: S8 Table — (DOCX) [file pone.0170084.s008.docx]

**S8 Table. Numbers and proportions of breast cancer clinicopathological subtypes of breast cancer patients with diabetes (type 1 and type 2) treated with or without insulin in subgroups of menopausal status.**

|  | **Premenopausal women with breast cancer** | | | | | | |  | **Postmenopausal women with breast cancer** | | |
| --- | --- | --- | --- | --- | --- | --- | --- | --- | --- | --- | --- |
|  | **Diabetes with Insulin**^*^ (n=34) |  | **Type 1 Diabetes with Insulin** ^*^ (n=19) |  | **Type 2 Diabetes with insulin** ^*^ (n=15) |  | **Diabetes without Insulin** ^†^ (n=76) |  | **Diabetes with Insulin** ^*^ **(**n=19) |  | **Diabetes without Insulin** ^†^ **(**n=82) |
| **Tumor subtype** | **% (n)** |  | **% (n)** |  | **% (n)** |  | **% (n)** |  | **% (n)** |  | **% (n)** |
| Grade 1 | 29.4 (10) |  | <11 (<5) ^ǂ^ |  | 53.3 (8) |  | 20.0 (15) |  | <19 (<5) ^ǂ^ |  | 16.9 (13) |
| Grade 2 | 41.2 (14) |  | 42.1 (8) |  | 40.0 (6) |  | 29.3 (22) |  | 31.3 (5) |  | 40.3 (31) |
| Grade 3 | 29.4 (10) |  | 47.4 (9) |  | <7 (<5) ^ǂ^ |  | 50.7 (38) |  | 50.0 (8) |  | 42.9 (33) |
|  |  |  |  |  |  |  |  |  |  |  |  |
| ER+ | 82.4 (28) |  | 73.7 (14) |  | 93.3 (14) |  | 70.7 (53) |  | 79.0 (15) |  | 81.7 (67) |
| ER- | 17.7 (6) |  | 26.3 (5) |  | <7 (<5) ^ǂ^ |  | 29.3 (22) |  | <22 (<5) ^ǂ^ |  | 18.3 (15) |
| PR+ | 76.5 (26) |  | 73.7 (14) |  | 80.0 (12) |  | 59.2 (45) |  | 63.2 (12) |  | 64.6 (53) |
| PR- | 23.5 (8) |  | 26.3 (5) |  | <21 (<5) ^ǂ^ |  | 40.8 (31) |  | 36.8 (7) |  | 35.4 (29) |
| HER2+ | <5 (<5) ^ǂ^ |  | <6 (<5) ^ǂ^ |  | - |  | 11.8 (9) |  | 13.5 (7) |  | 11.0 (9) |
| HER2- | 96.9 (31) |  | 94.4 (17) |  | 100 (14) |  | 88.2 (67) |  | 86.5 (45) |  | 89.0 (73) |
| Low ki67 | 60.6 (20) |  | 47.4 (9) |  | 78.6 (11) |  | 45.3 (34) |  | 63.5 (33) |  | 59.3 (48) |
| High ki67 | 39.4 (13) |  | 52.6 (10) |  | <22 (<5) ^ǂ^ |  | 54.7 (41) |  | 36.5 (19) |  | 40.7 (33) |

Missing values are not shown, therefore the sum of the categories does not add up to the total number of patients for grade, ER, HER2, ki67. * Women with diabetes treated with insulin (analogues) regardless the use of concomitant non-insulin antidiabetic drugs, † women with diabetes treated only with diet and exercise and users of non-insulin antidiabetic drugs only, ^ǂ^ exact numbers <5 with percentages cannot be shown according to regulations of Statistics Denmark.
