# Supplementary material for: Diabetes and Breast Cancer Subtypes
Source: PLoS One. 2017 Jan 11;12(1):e0170084. doi: 10.1371/journal.pone.0170084 (PMC5226802; doi:10.1371/journal.pone.0170084)
Supplement: S9 Table — (DOCX) [file pone.0170084.s009.docx]

**S9 Table. Crude odds ratios for** **breast cancer clinicopathological subtypes of premenopausal women with type 1 or type 2 diabetes treated with insulin compared to women without diabetes using (multinomial) logistic regression.**

| **Premenopausal women with breast cancer** | | | | | | | |
| --- | --- | --- | --- | --- | --- | --- | --- |
|  | **Independent variable of exposure** | | | | | | |
|  | **Type 1 Diabetes with Insulin** ^*^ **vs. No Diabetes** | |  | **Type 2 Diabetes with Insulin** ^†^ **vs. No Diabetes** | |  | **Diabetes only**  **Type 1 vs. Type 2 with Insulin** |
| **Dependent variable** | **crude OR (95% CI)** | **P** |  | **crude OR (95% CI)** | **P** |  | **P** |
| Grade 2 (vs. grade 1) | 1.57 (0.28-8.83) | 0.61 |  | 0.29 (0.08-1.09) | 0.07 |  | 0.08 |
| Grade 3 (vs. grade 1) | 2.38 (0.42-13.47) | 0.33 |  | **0.07 (0.01-0.62)** | **0.02** |  | **0.01** |
|  |  |  |  |  |  |  |  |
| ER- (vs. ER+) | 2.56 (0.68-9.69) | 0.17 |  | 0.51 (0.06-4.63) | 0.55 |  | 0.17 |
| PR- (vs. PR+) | 1.59 (0.45-5.55) | 0.47 |  | 1.11 (0.26-4.77) | 0.89 |  | 0.67 |
| HER2- (vs. HER2+) | 4.92 (0.59-41.22) | 0.14 |  | NE | NE |  | 0.96 |
| High ki67 (vs. low ki67) | 1.36 (0.47-3.94) | 0.57 |  | 0.34 (0.08-1.35) | 0.12 |  | 0.08 |

Logistic regression for tumor subtypes with 2 categories and multinomial logistic regression for tumor subtype with >2 categories as the dependent variable. * Women with type 1 diabetes treated with insulin (analogues) regardless the use of concomitant non-insulin antidiabetic drugs, † women with type 2 diabetes treated with insulin (analogues) regardless the use of concomitant non-insulin antidiabetic drugs. *OR=Odds Ratio, CI=Confidence Interval, NE= Not Estimated.*
