# Supplementary material for: Diabetes and Breast Cancer Subtypes
Source: PLoS One. 2017 Jan 11;12(1):e0170084. doi: 10.1371/journal.pone.0170084 (PMC5226802; doi:10.1371/journal.pone.0170084)
Supplement: S10 Table — (DOCX) [file pone.0170084.s010.docx]

**S10 Table. Adjusted odds ratios for breast cancer clinicopathological subtypes of women with diabetes treated with or without insulin compared to women without diabetes in subgroups of menopausal status using (multinomial) logistic regression.**

| **Premenopausal women with breast cancer** | | | | | | | |
| --- | --- | --- | --- | --- | --- | --- | --- |
|  | **Independent variable of exposure** | | | | | | |
|  | **Insulin ^*^ vs. No Diabetes** | |  | **No Insulin** ^†^ **vs. No Diabetes** | |  | **Diabetes only**  **Insulin vs.**  **No Insulin** |
| **Dependent variable** | **adjusted OR (95% CI)** | **P** |  | **adjusted OR (95% CI)** | **P** |  | **P** |
| Grade 2 (vs. grade 1) | 0.55 (0.18-1.68) | 0.29 |  | 0.58 (0.21-1.59) | 0.29 |  | 0.99 |
| Grade 3 (vs. grade 1) | 0.52 (0.15-1.77) | 0.30 |  | 1.52 (0.53-4.37) | 0.44 |  | 0.07 |
|  |  |  |  |  |  |  |  |
| ER- (vs. ER+) | 1.55 (0.45-5.38) | 0.49 |  | 2.86 (0.97-8.41) | 0.06 |  | 0.24 |
| PR- (vs. ER+) | 1.39 (0.47-4.10) | 0.55 |  | **2.70 (1.05-6.96)** | **0.04** |  | 0.13 |
| HER2- (vs. ER+) | **8.98 (1.09-74.19)** | **0.04** |  | 2.11 (0.71-6.25) | 0.18 |  | 0.20 |
| High ki67 (vs. low ki67) | 0.79 (0.32-1.99) | 0.62 |  | 1.46 (0.63-3.39) | 0.37 |  | 0.15 |

| **Postmenopausal women with breast cancer** | | | | | | | |
| --- | --- | --- | --- | --- | --- | --- | --- |
|  | **Independent variable of exposure** | | | | | | |
|  | **Insulin ^*^ vs. No Diabetes** | |  | **No Insulin** ^†^ **vs. No Diabetes** | |  | **Diabetes only**  **Insulin vs.**  **No Insulin** |
| **Dependent variable** | **adjusted OR (95% CI)** | **P** |  | **adjusted OR (95% CI)** | **P** |  | **P** |
| Grade 2 (vs. grade 1) | 0.53 (0.10-2.72) | 0.44 |  | 0.83 (0.31-2.19) | 0.70 |  | 0.91 |
| Grade 3 (vs. grade 1) | 1.91 (0.39-9.41) | 0.43 |  | 1.96 (0.69-5.58) | 0.21 |  | 0.81 |
|  |  |  |  |  |  |  |  |
| ER- (vs. ER+) | 1.65 (0.40-6.73) | 0.49 |  | 1.36 (0.50-3.66) | 0.55 |  | 0.64 |
| PR- (vs. ER+) | 1.10 (0.34-3.53) | 0.87 |  | 1.01 (0.47-2.17) | 0.99 |  | 0.94 |
| HER2- (vs. ER+) | 1.54 (0.21-11.28) | 0.67 |  | 1.13 (0.36-3.49) | 0.84 |  | 0.86 |
| High ki67 (vs. low ki67) | 0.74 (0.22-2.42) | 0.61 |  | 1.18 (0.55-2.51) | 0.67 |  | 0.49 |

Logistic regression for tumor subtypes with 2 categories and multinomial logistic regression for tumor subtype with >2 categories as the dependent variable. ORs were adjusted for age and BMI (continuous), except for grade which is adjusted for age only. * Women with diabetes treated with insulin (analogues) regardless the use of concomitant non-insulin antidiabetic drugs, † women with diabetes treated only with diet and exercise and users of non-insulin antidiabetic drugs only. *OR=Odds Ratio, CI=Confidence Interval.*
